# Orbitocranial Penetrating Injury With Multiple Vessel Invasion in an Infant: A Case Report and Literature Review

**DOI:** 10.3389/fneur.2020.591431

**Published:** 2020-11-12

**Authors:** Yun Wu, Tiange Chen, Meng Yuan, Juma Magogo Mzimbiri, Ziyuan Liu, Yilei Chen, Xiangying Luo, Fenghua Chen, Jinfang Liu

**Affiliations:** ^1^Department of Neurosurgery, Xiangya Hospital of Central South University, Changsha, China; ^2^Center for Experimental Medicine, The Third Xiangya Hospital of Central South University, Changsha, China; ^3^Muhimbili Orthopedic Institute, Dar Es Salaam, Tanzania

**Keywords:** case report, orbitocranial penetrating injury, multiple vessel invasion, infant, imaging, individualized strategy

## Abstract

Orbitocranial penetrating injury (OPI) with multiple vascular invasions is a rare occurrence. To our knowledge, experience with its clinical treatment is rather limited, especially for infants. This case report describes an infant who fell from a 0.5 m high bed and landed on a toy with a keen-edged plastic rod. The fractured end of the rod was noted at the medial aspect of the left eyelid, and she was experiencing impaired consciousness. Computed tomography showed that the foreign body penetrated the cavernous sinus with internal carotid artery involvement, and compressed the transverse sinus through the cerebellum. Emergency surgery was performed with temporal occlusion of the left common carotid artery. The rod was removed from the orbital side, and bleeding from cavernous sinus region was effectively controlled under direct inspection via a sub-temporal approach. The patient was successfully treated and recovered consciousness after 17 days. This is the first report of successful management of OPI combined with multiple vascular injury in an infant. Herein, we highlight the anatomical imaging features of the injuries and also the individualized strategy concerning vascular invasion.

## Introduction

Orbitocranial penetrating injury (OPI) refers to trans-orbital penetrating brain injury (PBI). PBI is significantly less prevalent than closed traumatic brain injury (TBI), with high mortality and morbidity rates (1–4). The complexity of PBI is related to various factors, including the site of injury, vascular involvement, and extent of penetration, which may lead to an elevated risk of subsequent operations. Orbitocranial penetration combined with multiple vessel injuries is an exceptionally rare neurosurgical emergency. Currently, there is no standardized protocol for PBI or OPI surgery. In particular, surgeries mostly rely on judgments based on experience and literature presented. For decades, the main objective of these operations has been to remove the foreign body (FB) in a timely, absolute, and effective manner (5–7). However, it should be noted that the removal process can worsen vessel injuries, which may result in hemorrhage, edema, and neuronal dysfunction. In this paper, we report an OPI with complex vessel injuries in an infant, where we avoided catastrophic sinus bleeding and prevented internal carotid artery (ICA) rupture by employing individualized strategies.

## Case Description

### Clinical Presentation

A 1-year 3-month-old girl was admitted to our hospital on August 29, 2019, 3 h after sustaining an OPI where a plastic rod pierced her orbital cavity via the lower left eyelid and penetrated the brain. The patient had no previous medical, family, or neurological disease history. Physical examination revealed that the patient was experiencing impaired consciousness, with a Glasgow Coma Scale score of 9. There was neither bleeding in the nasal cavity nor in the auditory canal. While the right pupil was 2 mm and sensitive to light, the left pupil was dilated and did not react to light. Movement in the right eye was normal; however, the left eye did not move. The patient demonstrated soft neck, and negative Kernig's and Brudzinski's signs. The right limbs were unresponsive to pain and the left limbs exhibited an avoidance reaction.

### Imaging Studies

**Figure 1:**
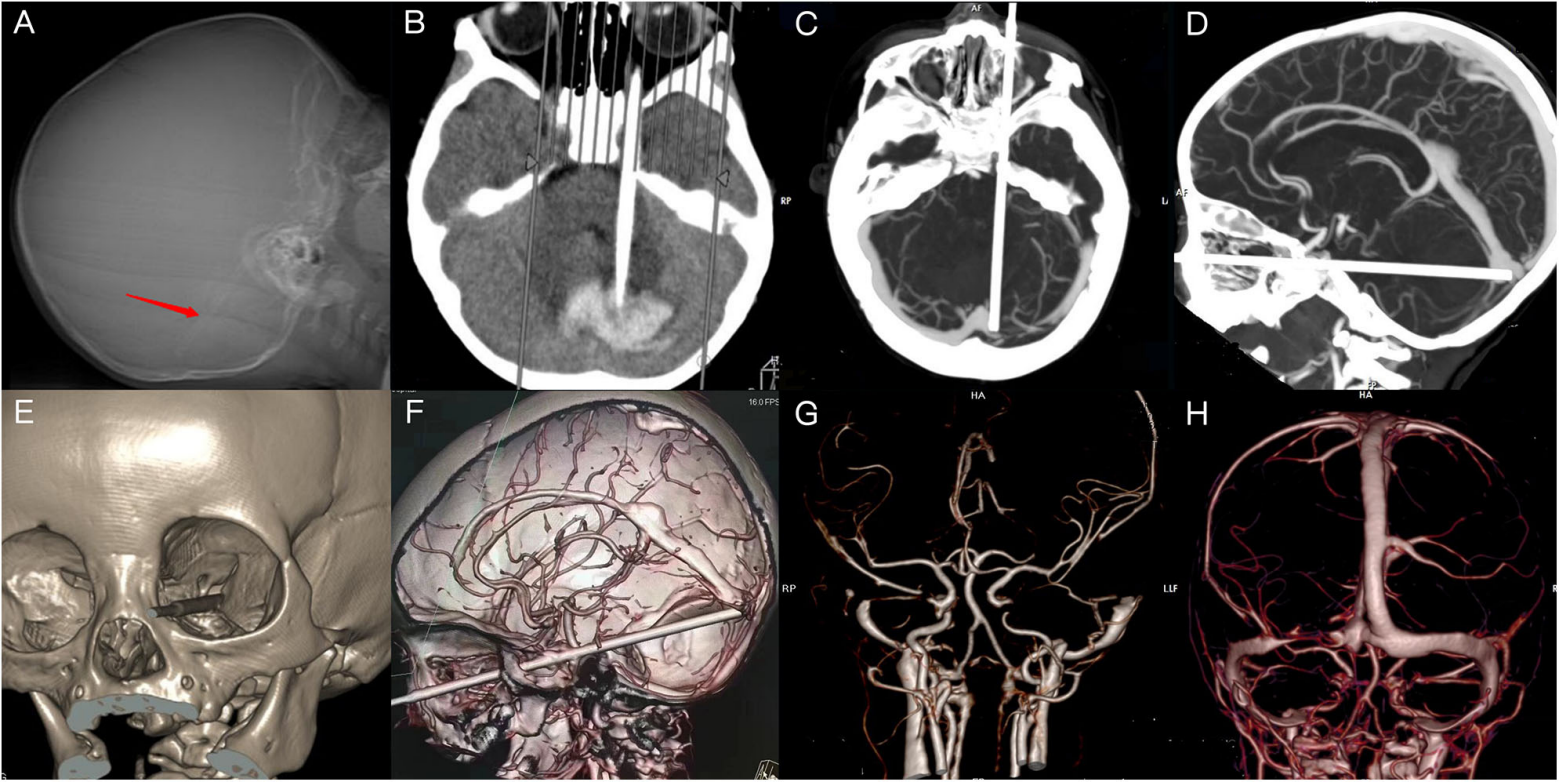
Imaging of X-ray and computed tomography (CT). **(A)** X-ray indicating the presence and position of a linear foreign body (FB). **(B)** CT revealing a strip-shaped focus and high-density areas in the adjacent areas. **(C,D)** The FB had pierced the left orbital apex and compressed the left transverse sinus. **(E,F)** The FB had penetrated the superior orbital fissure and cavernous sinus, in close proximity to the internal carotid artery (ICA). **(G)** Discontinuity of the cavernous segment of ICA. **(H)** Discontinuity in a portion of the left transverse sinus.

**Figure 2:**
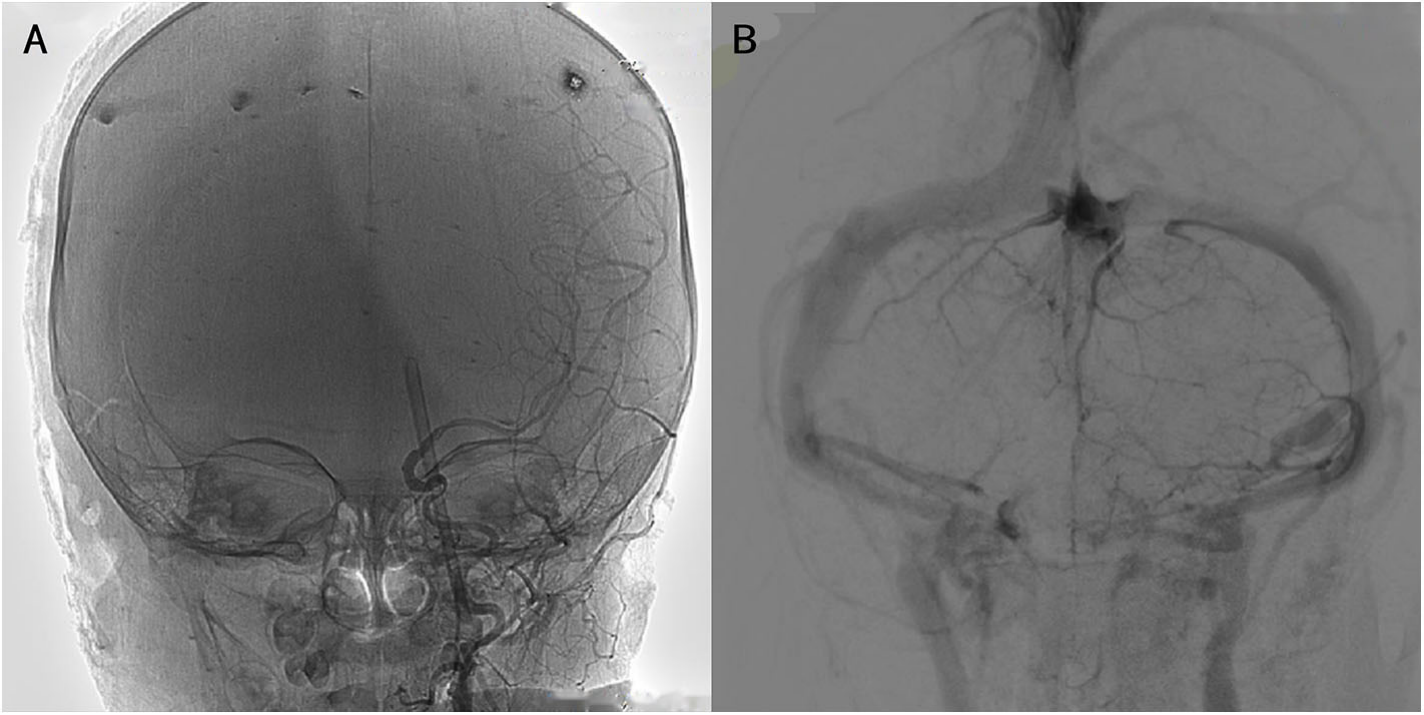
Imaging of digital subtraction angiography. **(A)** Focal compression of the C4 segment of the left internal carotid artery but no leakage of the contrast agent. **(B)** A poor developing transverse sinus with no leakage.

X-ray revealed an intracranial FB of ~130 mm in length. Fortunately, there was no sign of fracture. Computed tomography (CT) indicated that the FB had penetrated the medial, inferior portion of the eyeball; superior orbital fissure; parasellar region; circular cistern; and left cerebellar hemisphere. A hematoma was located in the cerebellar hemisphere and the cerebellar vermis, with edematous surrounding brain tissue and high density of the tentorium cerebelli. Three-dimensional CT reconstruction of the skull confirmed that the orbital wall was intact, with no sign of fracture or bone destruction. CT arteriography (CTA) revealed that the FB was close to the inner wall of the cavernous sinus segment of the left ICA. CT venography (CTV) showed that the tip of the FB was compressing the left transverse sinus (Figure 1). Digital subtraction angiography (DSA) suggested that compressive stenosis had occurred in the C4 segment of the left ICA with no sign of rupture, and the distal branches of left ICA were slightly sparse (Figure 2A). The left transverse sinus was also compressed but no rupture was observed (Figure 2B). The left ICA area was compensated by the right ICA via the anterior communicating artery and left posterior cerebral artery via the posterior communicating artery.

### Surgical Management

**Figure 3:**
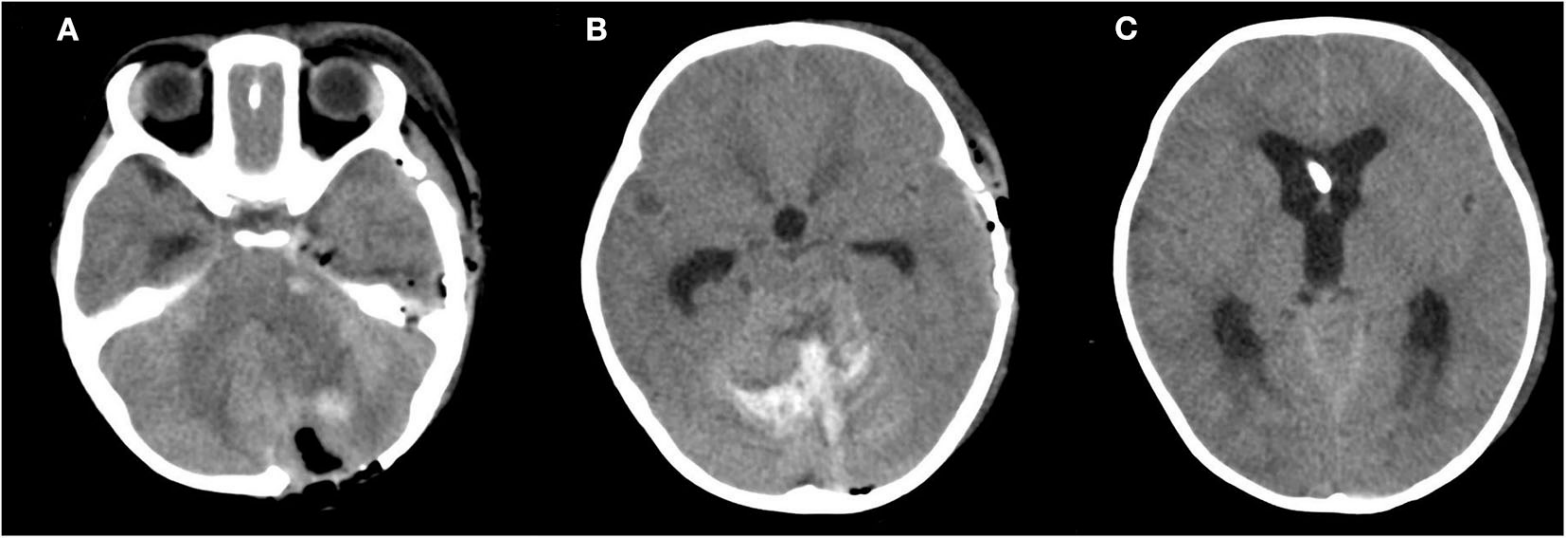
Postoperative computed tomography (CT). **(A,B)** CT scan revealing postoperative changes and high-density shade in the areas of the cavernous sinus region and cerebellum. **(C)** A high-density focus in the lateral ventricle.

The patient received emergency surgery on August 9, 2019. We first implanted an intracranial pressure (ICP) monitoring probe in the frontal horn of the right ventricle (initial pressure 15 mmHg). A straight incision was then made in the neck to fully expose the left common carotid artery (CCA) for temporary occlusion. Subsequently, the left cavernous sinus and part of the FB were exposed via a subtemporal approach. The FB was determined to have perforated the left cavernous sinus but had only damaged the ICA in a limited manner. To be cautious, we temporarily clipped the left CCA and then removed the rod from the side of the orbital socket. Because the ICA had not ruptured, bleeding from the cavernous sinus was soon staunched via electrocoagulation and compression. Fourteen hours after the first surgery, the ICP fluctuated around 25 mmHg, and a further CT scan revealed increased hemorrhage in the cerebellum. Therefore, we conducted a second surgery for cerebellar hematoma evacuation and removed ~5 mL of epidural hematoma from the left occipital region and ~14 mL of hematoma from the left cerebellar hemisphere. Postoperative CT demonstrated changes following hematoma evacuation and complete FB removal. The ICP probe maintained proper drainage and was correctly positioned in the lateral ventricle (Figure 3).

### Post-operative Management and Follow-Up

**Figure 4:**
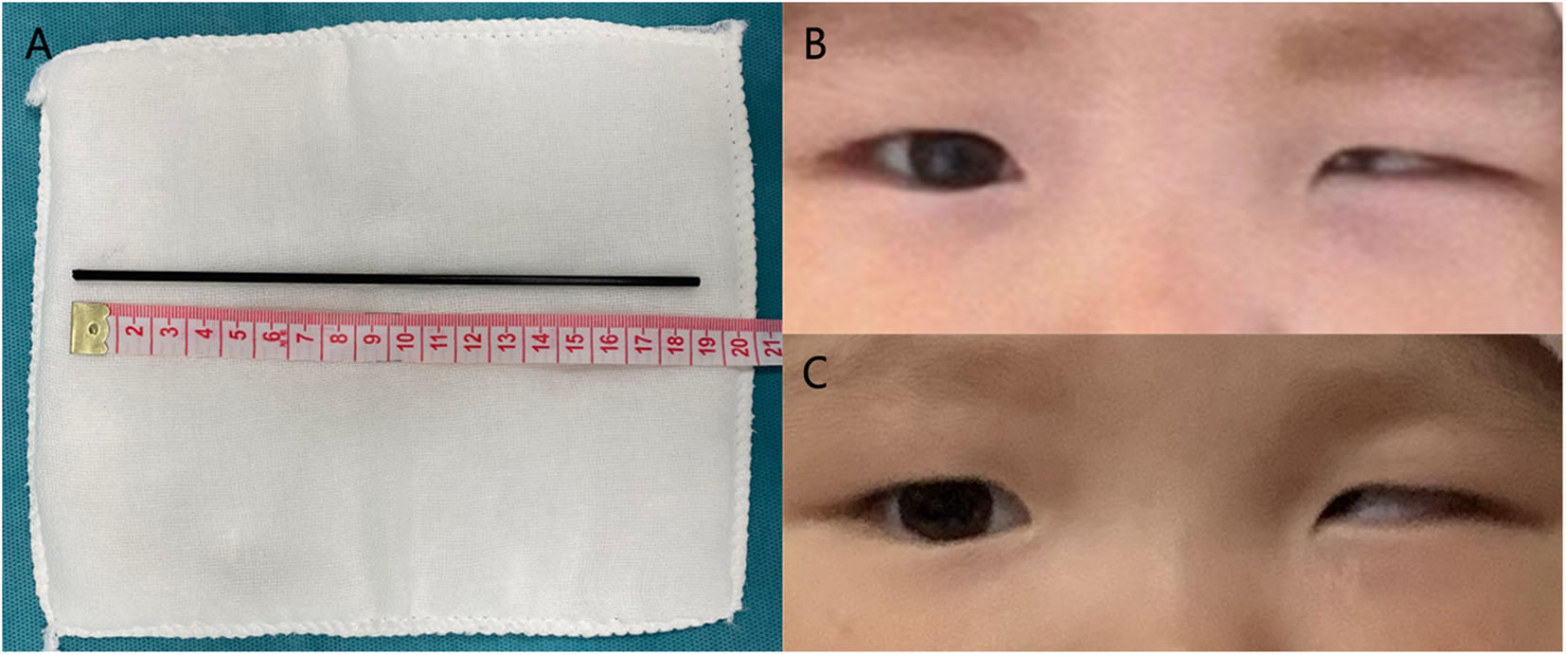
Postoperative photos. **(A)** Surgical removal of the plastic rod and appearance of both eyelids and palpebral fissures **(B)** Status at discharge. **(C)** Status at 3 months after surgery.

The FB was completely removed (Figure 4A). Postoperatively, the infant was transferred into the Neurological Intensive Care Unit. Primary postoperative treatments included ICP management, vasodilator therapy, anti-epilepsy treatment, lumbar puncture for hemorrhage drainage, and infection prevention with broad-spectrum antibiotics for 7 days. The state of illness gradually improved with symptomatic treatment, hyperbaric oxygen, and functional rehabilitation. The patient regained consciousness 17 days after surgery. At the time of discharge, she fully regained her consciousness, but still exhibited eye adduction, blepharoptosis (the eyelid fissure 4 mm), and dilated pupil with the absence of direct and indirect light reflexes on the left side (Figure 4B). When reexamined 3 months after discharge, the patient received a score of 5 on the Glasgow Outcome Scale, a tool intended for assessment in younger children. She exhibited normal movement and muscle strength in all four limbs. Due to non-compliance, we were unable to acquire a patient perspective and conduct an eyesight test. However, her left eye still exhibited blepharoptosis, adduction, limited outreach, and mydriasis. The left drooped eyelid fissure measured 6 mm across, but had improved and was slightly narrower than the right fissure (Figure 4C). One-year follow-up indicated she was GOS 5 with normal physical activity but still had blepharoptosis, adduction, limited outreach, and mydriasis, the chronic deficits resulting from OPI.

## Discussion

OPI is a subset of PBI, which is the most life-threatening form of TBI and can be classified into two types; high-energy and low-energy (6, 8, 9). In clinical practices, the majority of non-missile PBI are low-energy and commonly observed in the anatomically thin areas of the cranium, such as the squamous bones, basilar foramen, and orbital bones (10). The two types differ markedly in terms of impact velocity, trajectory, pathologic change, and clinical features (4). As with this case, OPI of low-energy results in direct lacerations and contusion of brain tissue, without the blast wave effect of bullets. In OPI, FBs are inclined to perforate the thin bone of the orbital roof and thus injure the frontal lobes (2). They can also penetrate the channels in orbital sockets, such as the superior orbital fissure and optic canal. When penetrating the superior orbital fissure, FBs tend to damage the cranial nerve (CN) III, IV, V, and VI and then extend to the cavernous sinus and brainstem (11, 12). In our infant patient, it was difficult to assess trigeminal nerve-related symptoms such as anesthesia of the forehead. However, the patient exhibited oculomotor palsy and deficiency of the trochlear and abductor nerves, which is a manifestation of traumatic superior orbital fissure syndrome (Figures 4B,C) (13, 14). In comparison, FBs near the optic nerve or the ICA are more likely to enter the suprasellar cistern directly (8). OPI is always complicated by multiple intracranial injuries, which emphasizes the need for thorough physical and imaging examinations.

Imaging plays a significant role in the choice of treatment strategy for OPI. X-ray is typically used for preliminary assessment and localization of intracranial FB. However, CT examination is the gold standard for the diagnosis of TBI and can be applied in OPI of different FB materials (15). MRI is normally used in the postoperative evaluation of diffuse axonal injury, brain ischemia, and brain swelling. However, MRI should be used with caution upon admission, because it can be time consuming and can cause complications in patients with metal FBs (6). The final test is DSA, which is invasive but enables physicians to assess the blood supply and subtle vascular injuries, which can be overlooked on CT (16, 17). In this case, CTA failed to show the precise anatomical relation between the FB and the ICA; we thus employed DSA and identified focal compression of the C4 segment of the ICA (Figure 2). Some reports have suggested that patient prognosis might be associated with vascular compensation and brainstem involvement, which can also be identified by imaging (18, 19). Further, aside from imaging diagnosis, ICP monitoring can provide useful guidance for planning secondary exploratory operations (such as in this case). There are few studies concerning ICP monitoring in OPI or PBI, but this could be a field worthy of further investigation.

There is currently no standardized OPI guideline. However, treatment principles coincide with other PBI subtypes, including complete FB removal, local debridement, neurovascular decompression, hemostasis, and dural repair (10). Early craniotomy is warranted in most cases. Before surgery, it is necessary to clearly define the material and integrity of FBs, anatomic injury features, and its effects on blood flow. During surgery, FB removal may lead to excessive bleeding due to loss of tamponade. Therefore, it is essential to make preparations directed by potential vascular complications. Based on FB location and associated intracranial complications, a transorbital or transcranial approach can be utilized (2, 20, 21). Our previous studies reported several typical transcranial approaches, encompassing pterional, subfrontal, and subtemporal craniotomy. However, specific operations were determined by clinical findings. Temporary or permanent vessel occlusion should be considered in cases where major vascular injury exists with high risk of excessive bleeding (2). In our case, rapid hemostasis was achieved via a subtemporal approach, which enabled visual inspection of the left ICA and the cavernous sinus. Cerebral vascular function in our patient was compensated efficiently because FB removal alleviated compression of the transverse sinus. Moreover, there are abundant communicating branches between the cavernous sinus and cerebral veins. Temporary clip occlusion of the left CCA was a precaution for fatal bleeding in this patient, but it could be an instructive reference for other lethal vascular conditions. Due to the technological limitations at that time, we could perform only DSA in the hybrid operating room. As an effective and low-injury means of vascular occlusion, endovascular techniques can be a better solution than CCA exposure, such as temporary balloon occlusion or stent implantation (22).

OPI outcome is generally good in absence of direct injury to the brainstem or laceration of major vessels (23). Quite a few patients might be left with sequelae such as extraocular movement disorders, ptosis, and loss of vision (24). Consistent with other PBI subtypes, OPI tends to develop systemic complications, particularly intracranial complications. Infant patients are more vulnerable to severe complications due to poor tolerance to trauma (25). The most common intracranial complication is intracranial hemorrhage. Other complications include infection, CN injuries, ischemia, pseudoaneurysm formation, arteriovenous fistula, venous sinus occlusion, epilepsy, hydrocephalus, and cerebrospinal fluid leakage (1, 6, 26, 27) The incidence of intracranial hemorrhage is 31–78% and the prognosis is poor (28, 29). In addition, there is a high risk of intracranial infection in PBI patients, possibly due to contamination by FBs. Monitoring infection indicators and early administration of prophylactic antibiotics for ~7–14 days are suggested (28). Rapid FB removal would reduce the incidence of postoperative infection. However, any residual FBs may cause severe infectious complications, such as brain abscesses, for which the mortality rate is >50% (18, 28, 30). Nevertheless, this patient currently has no signs of intracranial infection or meningitis, which may be attributed to the active use of antibiotics, timely operation, and complete FB removal.

OPI poses tough challenges to neurosurgeons worldwide due to the anatomical complexity of the trans-orbital pathway. For diagnosis, clinicians have to simultaneously consider FBs, and injury to the eyes, orbit, CNs, vessels, FBs, and brain tissues. This implies a greater requirement of diagnostic techniques, including high-resolution CT, MRI, and DSA (31, 32). For treatment, there are currently no standardized guidelines, especially in advanced techniques such as endovascular treatment, temporary artery occlusion and ICP monitoring. Application of these techniques, mainly as case studies, still requires systemic research (25, 33). Lastly, management of disciplinary integration for complications is still a long way ahead (21).

## Conclusion

In this report, we highlight the importance of assessment based on CT reconstruction, CT angiography, and DSA, which are crucial for reference in OPI complicated with vascular injuries. The key part of an OPI surgery is decompression and removal of FBs, while preserving the normal tissue as much as possible. As an individualized strategy, temporary vessel occlusion is a choice when there is the possibility of lethal vascular damage. Infant cases put forward significant demand for perioperative management, particularly the control of complications. These treatment processes require close cooperation among multidisciplinary teams, involving neurotrauma, neuroimaging, ophthalmology, and neurocritical care.

## Data Availability Statement

The original contributions presented in the study are included in the article/Supplementary Materials, further inquiries can be directed to the corresponding author/s.

## Ethics Statement

Ethical review and approval was not required for the study on human participants in accordance with the local legislation and institutional requirements. Written informed consent to participate in this study was provided by the participants' legal guardian/next of kin. Written informed consent was obtained from the individual(s), and minor(s)' legal guardian/next of kin, for the publication of any potentially identifiable images or data included in this article.

## Author Contributions

YW: contribution to the conception, draft and revision of the work, analysis, acquisition, and interpretation of data. TC: contribution to the acquisition of data and revision of the work. MY, JM, ZL, and YC: contribution to the revision of the work and interpretation of data. XL: contribution to the conception and revision of the work. FC: contribution to the enlightenment of treatment strategy. JL: conception, revision of the work and analysis, acquisition, and interpretation of data. YW, TC, MY, JM, ZL, YC, XL, FC, and JL: approval of the final manuscript as submitted, and agreement to be accountable for all aspects of the work. All authors contributed to the article and approved the submitted version.

## Conflict of Interest

The authors declare that the research was conducted in the absence of any commercial or financial relationships that could be construed as a potential conflict of interest.
