# Supplementary material for: Orbitocranial Penetrating Injury With Multiple Vessel Invasion in an Infant: A Case Report and Literature Review
Source: Front Neurol. 2020 Nov 12;11:591431. doi: 10.3389/fneur.2020.591431 (PMC7689382; doi:10.3389/fneur.2020.591431)
Supplement: Supplementary file 1 [file Data_Sheet_1.docx]

Supplement material: timeline of treatment process.
